# Selective uptake of epidermal growth factor-conjugated gold nanoparticle (EGF-GNP) facilitates non-thermal plasma (NTP)-mediated cell death

**DOI:** 10.1038/s41598-017-11292-z

**Published:** 2017-09-08

**Authors:** Wanil Kim, Kyung-Yoon Na, Kyung-Ha Lee, Hyun Wook Lee, Jae Koo Lee, Kyong-Tai Kim

**Affiliations:** 1 Department of Life Sciences, Pohang University of Science and Technology (POSTECH), Pohang, 37673 Republic of Korea; 2 Division of Integrative Biosciences and Biotechnology, Pohang University of Science and Technology (POSTECH), Pohang, 37673 Republic of Korea; 3 Division of Biotechnology and Convergence, Daegu Haany University, Gyeongsan, 38610 Republic of Korea; 4 Department of Electronic and Electrical Engineering, Pohang University of Science and Technology (POSTECH), Pohang, 37673 Republic of Korea; 5 R&D Unit, AmorePacific Corporation, Yongin, 17074 Republic of Korea

## Abstract

Non-thermal atmospheric pressure plasma (NTP) has been shown to induce cell death in various mammalian cancer cells. Accumulated evidence also shows that NTP could be clinically used in cancer therapy. However, the current NTP-based applications lack target specificity. Here, a novel method in NTP-mediated cancer therapeutics was described with enhanced target specificity by treating EGF (epidermal growth factor)-conjugated GNP (gold nanoparticle). The treatment with EGF-conjugated GNP complex, followed by NTP irradiation showed selective apoptosis of cells having receptor-mediated endocytosis. NTP triggered γ–H2AX elevation which is a typical response elicited by DNA damage. These results suggest that EGF-conjugated GNP functions as an important adjuvant which gives target specificity in applications of conventional plasma therapy.

## Introduction

Apoptosis is programmed and orchestrated cell death, and its primary role is to keep cell number homeostasis in multicellular organisms^1–4^. Apoptosis is not only important in normal embryonic development such as limb morphogenesis^5^, but also important in targeting of naturally occurring malignant cells^6^. Therefore many researchers tried to elicit apoptotic cell death in cancer cells with various strategies such as tubulin blockers or DNA damage-eliciting chemicals^7^. Additional specificity was conferred by using target-specific small molecule inhibitors, monoclonal antibodies, and nucleotides^8, 9^.

Added to these conventional cancer therapies, Fridman G *et al*. described “plasma medicine” that uses non-thermal atmospheric pressure plasma (NTP) to effectively remove cancer cells as well as to sterilize non-living objects^10^. NTP induced significant changes in mammalian cells including surface detachment of CHO-K1 and loss of cell-cell interaction^11^. NTP also induced DNA damage, followed by apoptotic cell death^12, 13^. Generation of reactive oxygen and nitrogen species are often attributed to the apoptotic responses of the NTP treatment^14^, but the detailed mechanism is still largely unknown. One of the important characteristics of the NTP is caner-cell specific cytotoxicity^15^. A recent report focused on cytotoxicity of NTP on p53-mutated cells, implying that cancer-specific genetic alterations might be responsible for the preferential cytotoxicity^16^. However, the detailed mechanism for this still awaits extensive studies.

Nanotechnology-coupled cancer therapy has also important roles in this field^17^. Injection of gold nanoparticle (GNP) into mice with xenografted EMT-6 mammary carcinoma cells, followed by x-ray therapies showed a significant delay in tumor growth^18^. Particularly, synergistic combination of GNP and NTP showed potential in improving cancer therapy^19, 20^. For target specificity, Kim *et al*. also showed that GNP-conjugated antibody against FAK (Focal adhesion kinase) protein effectively targets tumor and increases cell death after NTP irradiation^21^.

Since the EGFR (EGF Receptor) is a strong prognostic indicator in human epithelial cancers^22^, we prepared epidermal growth factor (EGF)-conjugated GNP and treated this to cancer cells which express a high level of EGFR. Here, we report that selective uptake of EGF-GNP complex, followed by NTP treatment efficiently triggered apoptosis. We observed receptor-mediated endocytosis of the complex. Treatment with NTP also induced a significant increase in apoptosis in the EGF-conjugated GNP complex-treated cells. Taken together, we suggest that the EGF-conjugated GNP complex coupled with NTP treatment efficiently targets EGFR-expressing cancer cells.

## Results

### Development of nonthermal air plasma (NTP)-generating device for cell treatment

**Figure 1:**
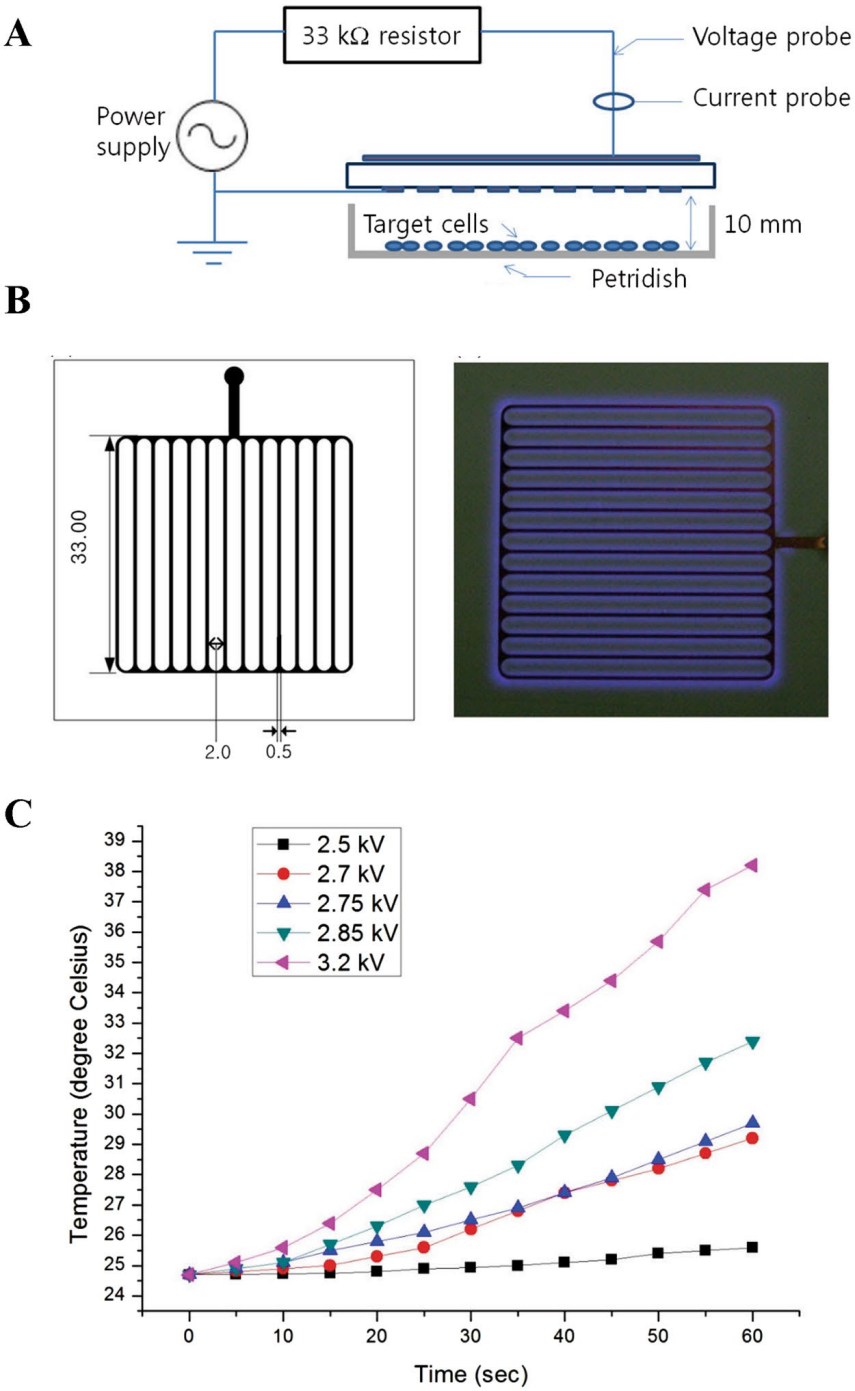
Development of nonthermal air plasma (NTP)-generating device for cell treatment. (**A**) The schematic diagram of the experimental setup. (**B**) The grounded electrode of the plasma source and light emission from the air plasma source. (**C**) The temperature measured at the plasma source of each driving voltage for 60 seconds.

To address the specific and differential effect of NTP on GNP-treated cells, we devised a NTP-irradiating system as we previously described^12^. Figure 1A shows a schematic diagram of the originally devised plasma irradiation system. Atmospheric pressure surface-type plasma source was developed to cover and treat whole target area. A polytetrafluorethylene (PTFE) dielectric (ε_r_ = 2.2, 750 μm thickness) with Cu electrode (35 μm thickness) on both sides was employed to manufacture the plasma source. The plasma source based on the device reported by *Kim et al*.^12^, had 3.3 cm by 3.3 cm striped mask pattern, and the pattern was engraved by a conventional etching method (Fig. 1B left panel). High voltage electrode on the back side of the plasma source was connected to a power source (15 kV maximum voltages, 22 kHz) through 33 k resistor. The striped electrode on the front side was grounded, and directed towards the sample. Micro-size filamentary discharge was generated and distributed uniformly around the grounded electrode (Fig. 1B right panel). The plasma source operated with voltages ranged from 2.5 kV to 3.2 kV magnitudes in ambient air, atmospheric pressure. The breakdown voltage of the plasma source was approximately 2 kV and the intensity of plasma was proportional to voltage. The temperature was measured at 10 mm distance from the plasma source, which was the same distance with the location of the cells. The maximum temperature was ~38 °C at 3.2 kV after 60 seconds exposure, while the temperature rarely raised at 2.5 kV (Fig. 1C). Even if we chose various driving voltages ranging from 2.5 kV to 3.2 kV, there was a little change in temperature which does not exceed physiological condition. The result shows that our device generates stable and safe plasma that could be applied clinically with no damage to cells. Approximately 1,000 ppm of ozone was produced by the air plasma as previously reported^12^. The filamentary discharge was generated consuming 4.27 W and energy density of about 20 J/cm^2^ was estimated for the 30 seconds of treatment as per our previous result.

### A549, human lung carcinoma cell, expresses high level of EGFR

**Figure 2:**
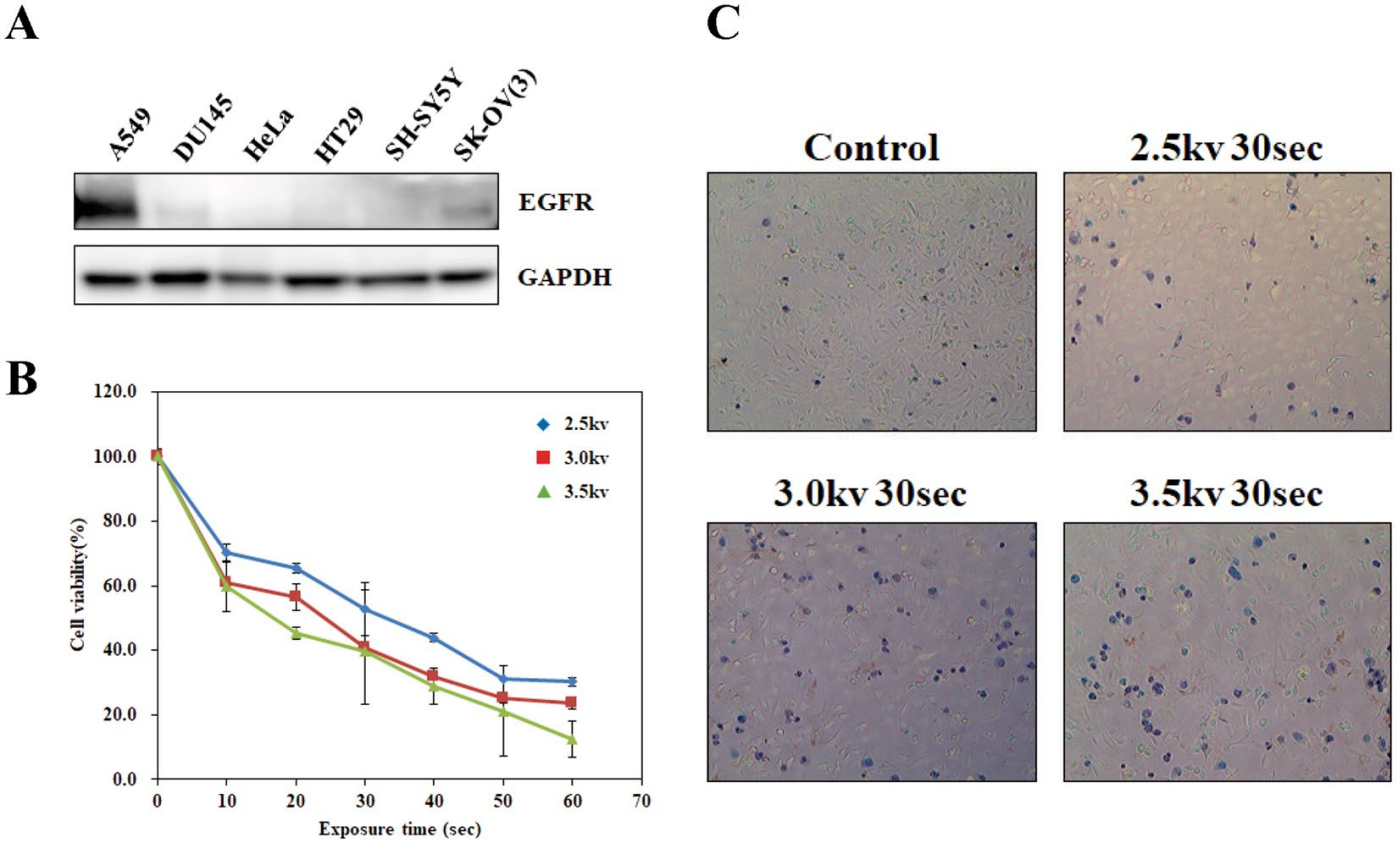
A549, human lung carcinoma cell, expresses a high level of EGFR. (**A**) A549, DU145, HeLa, HT29, SH-SY5Y, and SK-OV (3) cells were cultured and whole-cell extracts were analyzed by Western blotting for EGFR assessment. Glyceraldehyde-3-phosphate dehydrogenase (GAPDH) was used as a loading control. (**B**) A549 cells were cultured in 35 mm^2^ dishes until 80~90% of confluency. NTP was generated at different voltages and cells were exposed for different time periods as indicated in the figure. Each value represents the mean ± S.D. of triplicate samples. (**C**) Trypan blue staining was performed to assess dead cell population after plasma treatment. Exposure time and condition are indicated in the figure.

Mitogenic signal elicited by extracellular EGF (Epidermal Growth Factor) is one of the most prominent stimuli for cell proliferation. The signaling cascade passes through various proto-oncogenes containing EGF-responding receptor tyrosine kinases^23^. Particularly, ectopic expression of the EGF receptor (EGFR) on the cell surface is one of the main causes of carcinogenesis and the most well-known diagnostic marker for various malignant tumors^24^. Thus we determined to target EGFR-expressing cells by treating EGF-conjugated GNP since uptake of GNP was also known to induce apoptosis^18, 21^. To investigate the expression level of the EGFR in cancer cells, we performed Western blotting analysis of candidate cancer cells (Fig. 2A). We selected EGFR-positive cancer cell lines including A549 human lung carcinoma cell, DU145 human metastatic prostate cancer cell, HeLa human cervical carcinoma cell, HT29 human colorectal adenocarcinoma cell, SH-SY5Y human bone marrow neuroblastoma cell, and SK-OV(3) human ovarian carcinoma cell lines^25–29^. Among them, we selected the A549 non-small cell lung cancer fibroblast for further analyses because the cell showed the highest expression of the EGFR among the panel of the cancer cell lines. We also preferred the A549 cell line since the cell showed enhanced cytotoxicity in response to Cetuximab (C225)-conjugated gold nanoparticles in previous work^30^.

We next treated the NTP to the A549 cell to assess responses of the cell and effect of the NTP. The higher voltage induced an increase in cytotoxicity in both MTT analysis and Trypan blue staining analysis in a concentration-dependent manner (Fig. 2B and C). Taken together, the A549 cell expressing a high level of the EGFR was a proper model for our system to show apoptotic responses to the NTP.

### Preparation of the Epidermal Growth Factor (EGF)-conjugated gold nanoparticle (GNP)

**Figure 3:**
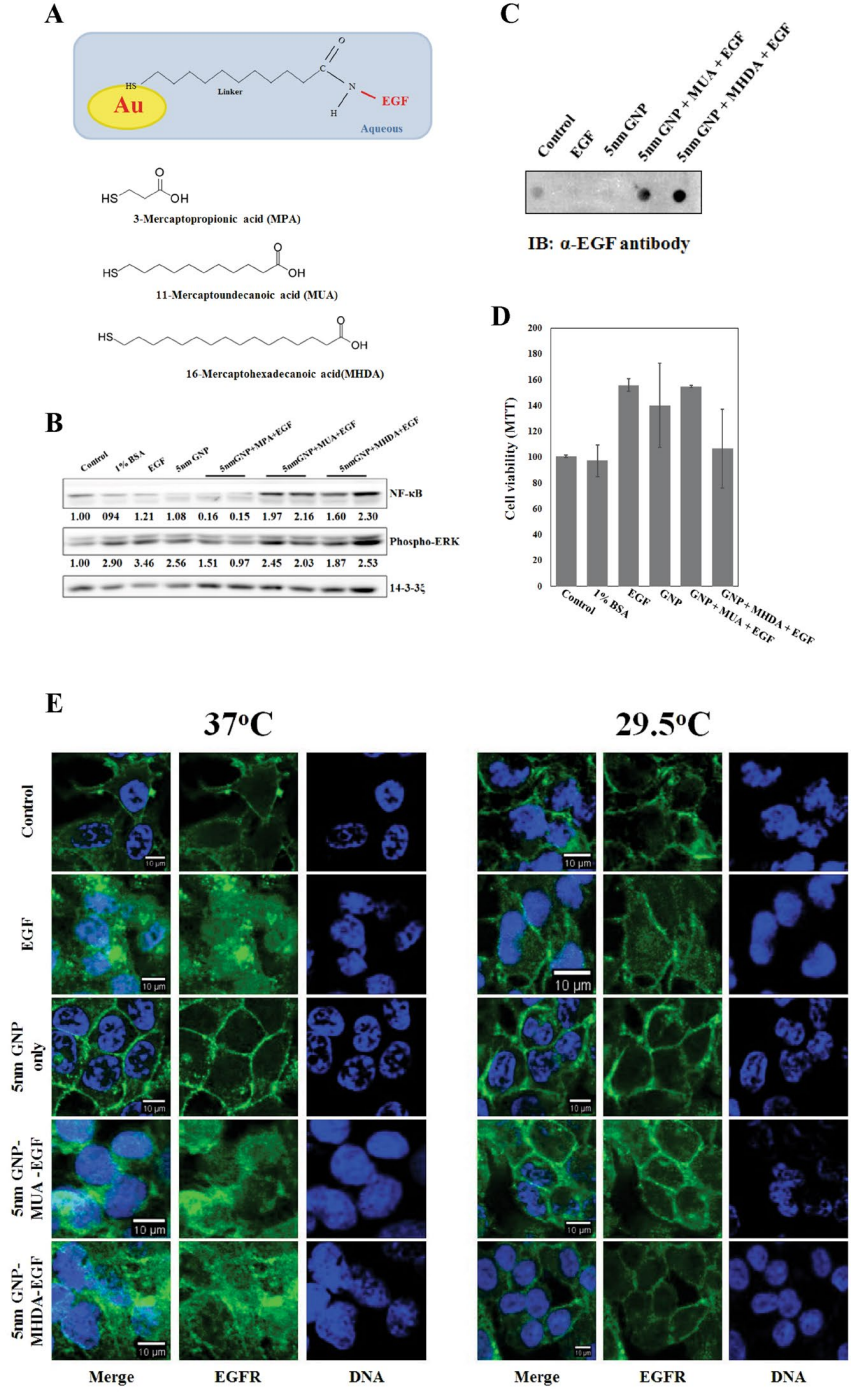
Preparation of the Epidermal Growth Factor (EGF) conjugated gold nanoparticle (GNP). (**A**) The experimental scheme shows conjugation between EGF and GNP. Three different linkers were used for conjugation; MPA (3-mercaptopropionic acid), MUA (11-mercaptoundecanoic acid), and MHDA (16-mercaptohexadecanoic acid) (**B**) Serum-starved (24 hours) A549 cells were treated with 1% BSA, EGF (100 μg/ml), 5 nm GNP, 5 nm GNP-MPA-EGF, 5 nm GNP-MUA-EGF, and 5 nm GNP-MHDA-EGF respectively. Whole-cell extracts were analyzed by Western blotting for assessment of the level of phospho-ERK protein. 14-3-3ξ was used as a loading control. Quantification of Western blots was performed with Image J. All density values were normalized with the loading control. (**C**) Whole-cell extracts were analyzed by dot blots. EGF antibody detected 5 nm GNP-MUA-EGF and 5 nm GNP-MHDA-EGF. The experiment was performed independently at least three times. (**D**) Serum-starved (24 hours) A549 cells were treated with 1% BSA, EGF (100 μg/ml), 5 nm GNP, 5 nm GNP-MUA-EGF, and 5 nm GNP-MHDA-EGF for 6 hours at 37 °C, respectively. MTT assay was performed to assess cell viability after incubation without plasma irradiation. (**E**) Serum-starved (24 hours) A549 cells were treated with EGF (100 μg/ml), 5 nm GNP, 5 nm GNP-MUA-EGF, and 5 nm GNP-MHDA-EGF, respectively. Cells were incubated at 37 °C or 29.5 °C for 6 hours. EGFR was stained with green fluorescent antibody, and DNA was stained with Hoechst 33342. The scale bar on each image represents 10 μm.

We proposed earlier that the NTP treatment with epidermal growth factor (EGF)-conjugated gold nanoparticle (GNP) could be a new approach in targeted cancer therapy. Thus we conjugated the EGF to the GNP with three different linkers; MPA (3-mercaptopropionic acid), MUA (11-mercaptoundecanoic acid), and MHDA (16-mercaptohexadecanoic acid) (Fig. 3A). Since propagation of EGF signaling activates extracellular signal-regulated kinases (ERKs)^31, 32^, we examined level of the phosphorylated ERK in conjugates-treated cells to validate activity of the conjugates. Treatment with the EGF alone showed a significant increase of the phospho-ERK (Fig. 3B). The conjugated EGF with MUA and MHDA also showed significant elevation of the phospho-ERK, but MPA conjugation had no effect in the treated cells. The A549 cell was also stimulated by treatment with 1% BSA and 5 nm GNP even though the mechanism is not known. However, it was previously reported that nanoparticles trigger activation of MAPK signaling including ERK, p38, and JNK for proliferation or inflammation^33^. We also expect that treatment with 1% BSA induced activation of the ERK signaling since it is already known that serum albumin interacts with a lot of biomolecules^34^. We further examined the expression of NF-κB since the signaling is closely associated with proinflammatory responses^35^. As shown in Fig. 3B, expression of the NF-κB was significantly increased by the treatment with the EGF-GNP complexes conjugated with MUA and MHDA. Since both ERK and NF-κB signaling were activated by the treatment with EGF-MUA-GNP and EGF-MHDA-GNP, we chose MUA and MHDA linkers for our further studies.

Dot botting analysis also showed that the conjugates were specifically recognized by the anti-EGF antibody (Fig. 3C). It is notable that we could not detect a significant amount of EGF staining when we treated cells with EGF alone. This might be due to internalized EGF was already degraded or was not persistent enough to trigger downstream signaling. This could be one of the reasons why the treatment with EGF could not induce NF-κB signaling although the level of the phospho-ERK was significantly increased (Fig. 3B). We expect that the GNP-conjugated EGF was retained more in the treated cells compared to unmodified EGF in transduction of downstream signaling.

We also assessed cell viability after the treatment with EGF-GNP conjugates without plasma irradiation (Fig. 3D). Treatment with EGF and GNP alone induced a subtle increase in cell viability, which might be triggered by the ERK signaling as we shown in Fig. 3B. The EGF-GNP conjugates also showed a subtle increase in cell viability, implying that upregulated NF-κB was not a key factor to elicit an apoptotic response without subsequent NTP irradiation.

It is well-established that the EGF triggers dimerization of the epidermal growth factor receptor (EGFR), followed by recruitment and activation of intracellular signal transducers such as proteins containing SH2 (src homology domain 2) or PTB (phosphotyrosine binding) domain^36, 37^. Ligand binding also triggers recruitment of the EGFR to clathrin-coated pits, and then the EGFR-ligand complex is internalized^32^. To examine that the EGF-conjugated GNPs move into the cell with the EGFR, we treated cells with the EGF-conjugated GNP and stained the EGFR protein (Fig. 3E). The result showed that the EGFR was internalized when we treated the cells with EGF or GNP-conjugated EGF at 37 °C. We also examined this at different temperature of 29.5 °C because receptor-mediated endocytosis is significantly affected by culture temperature^38^. Intracellular localization of the EGFR induced by the treatment with EGF or GNP-conjugated EGF was significantly diminished when we maintained the treated cells under 29.5 °C. The EGFR protein remained on the plasma membrane when the cells were treated with the GNP-conjugated EGF at low temperature. The result implies that the internalization of the conjugates might depend on the endocytotic pathway.

### Treatment with EGF-conjugated GNPs enhanced apoptotic response by NTP irradiation

**Figure 4:**
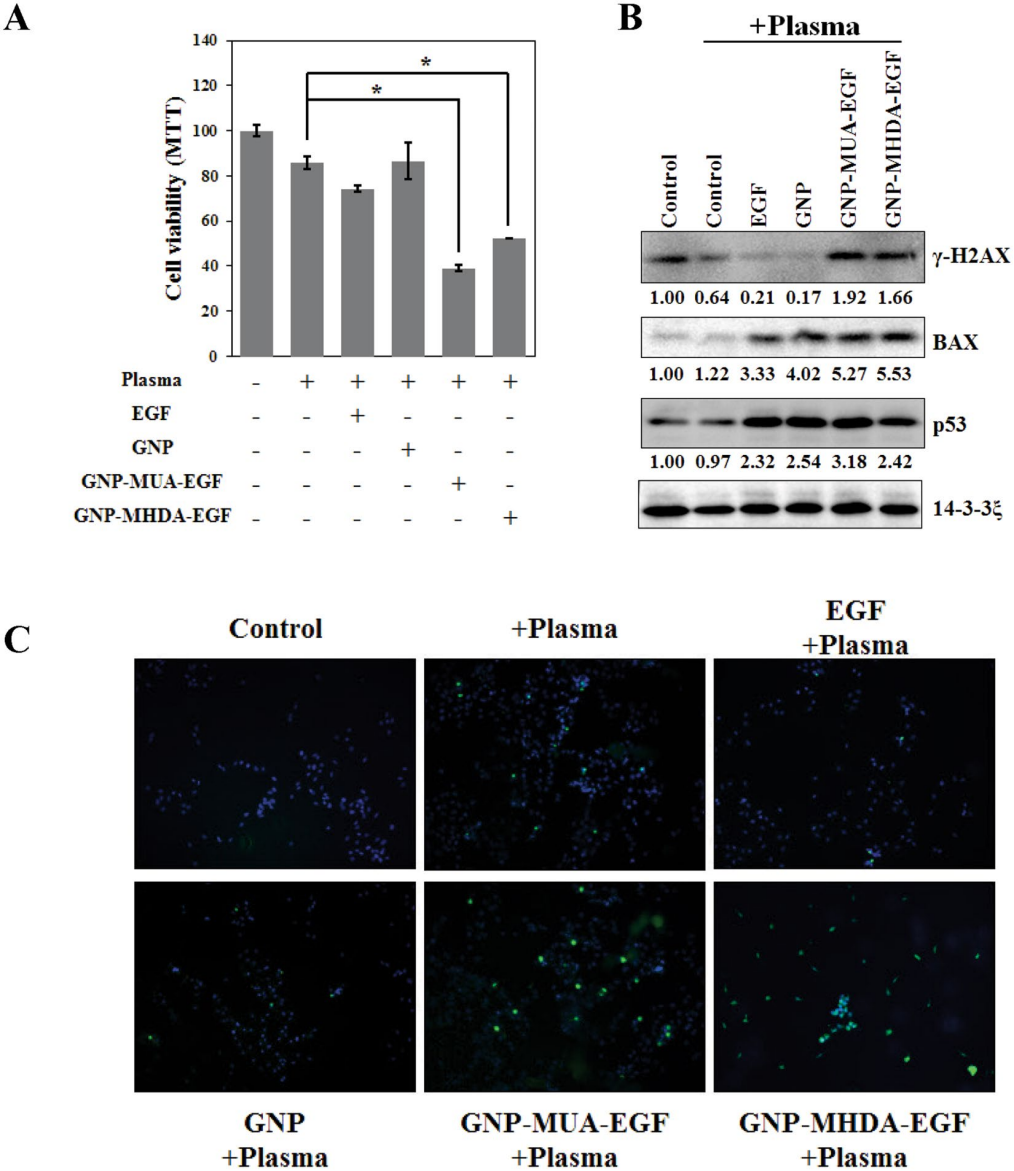
Treatment with EGF-conjugated GNPs enhanced apoptotic response by NTP irradiation. MTT assay shows cytotoxicity of NTP irradiation after GNP-EGF treatment. Serum-starved (24 hours) A549 cells were treated with EGF (100 μg/ml), 5 nm GNP, 5 nm GNP-MUA-EGF, and 5 nm GNP-MHDA-EGF for 6 hours at 37 °C, respectively. After NTP irradiation at 2.5 kV for 50 seconds, cells were incubated for 6 hours at 37 °C. (**A**) MTT assay was performed right after final incubation. Student t-test revealed significance. *p < 0.05 (**B**) Protein level of γ-H2AX was assessed by Western blotting after the GNP-EGF treatment, followed by NTP irradiation. 14-3-3ξ was used as a loading control. (**C**) TUNEL assay was performed to assess the cytotoxicity of NTP irradiation after GNP-EGF treatment.

Since cell-adjacent GNPs induced enhanced apoptosis as we previously reported^21^, we hypothesized that the endocytosed GNP would also effectively kill the treated cells. Thus we next assessed viability of the NTP-irradiated cells under treatment with the EGF-conjugates. MTT analysis was performed to examine apoptotic response of the cells after plasma irradiation (Fig. 4A). NTP treatment induced apoptosis in 20% of treated cell population. Treatment with EGF and GNP alone showed no significant changes. However, treatment with the EGF-conjugated GNP, followed by the NTP irradiation significantly increased dead cell population. This result shows that the internalized GNP responded to the NTP and effectively killed the cells.

We next examined if DNA damage was one of the causes for this response (Fig. 4B). We first analyzed expression of BAX (Bcl2-associated X protein) and p53 since they are well-known markers for apoptosis^39^. The protein level of BAX and p53 was significantly increased in the NTP-treated cell populations, suggesting that the treatment with the NTP induced mitochondria damage as we previously reported^12^. However, protein level of γ-H2AX was only significantly increased in cell populations treated with the GNP-EGF conjugates. The result suggests that DNA damage-dependent early apoptotic responses were elicited in the cells treated with GNP-EGF conjugate coupled with NTP. We further performed TUNEL assay to show that the treatment with the EGF-conjugated GNP facilitated NTP-induced cell death (Fig. 4C). Taken together, these data suggest that the EGF-conjugated GNP coupled with the NTP irradiation effectively induced apoptosis in the treated cells.

## Discussion

We hypothesized that receptor-mediated endocytosis of EGF-GNP conjugates could target EGFR-expressing cells and cause selective apoptosis by subsequent NTP irradiation. In this report, we have shown that our EGF-GNP conjugate elicited phosphorylation of ERK, suggesting that the complex is functional as a signaling mediator to stimulate EGFR. Internalization of the EGFR further suggested that gold nanoparticle internalized in the mammalian cell would effectively respond to NTP irradiation to increase apoptosis. We further showed that NTP irradiation coupled with EGF-GNP treatment triggered DNA damage response to induce cell death. Thus, we think that this novel strategy would be applied to treat cancer cells expressing higher level of the EGFR.

Nanotechnology was developed as one of the therapeutic approaches against cancer^17^. Application of GNP in the cancer therapy is increasing due to its unique perpetuity^40, 41^. Therefore, we tried to conjugate this to EGF for application in the plasma medicine. At first, we used three different linkers including MPA, MUA, and MHDA to make self-assembled monolayer (SAM) between EGF and GNP. We finally adopted MUA and MHDA linkers which are composed of eleven and sixteen carbons, respectively. We dropped MPA since it showed no receptor-mediated endocytosis of EGF-GNP conjugates, followed by no change in ERK signaling (Fig. 3B). The result suggests that short alkanethiols in SAM made with MPA do not have adequate space for a proper function.

We previously suggested that treatment with antibody-conjugated gold nanoparticle significantly enhances NTP-mediated cell death^42^. However, low cell permeability and inaccurate targeting due to steric hindrance from the large antibody molecule dampen the enthusiasm of this strategy. Thus, we propose our new strategy in this report by using the GNP-conjugated EGF ligand to target EGFR-expressing cancer cells for NTP irradiation. We think that gold nanoparticles penetrate into A549 cells without any modification through receptor-mediated endocytosis as there are a few reports for this^43, 44^. However, we thought that this process could be enhanced by tagging the gold nanoparticles with EGF ligand. We indirectly showed this by performing MTT analysis in Fig. 4A. Treatment with the GNP followed by NTP showed little apoptosis, but the EGF-conjugated GNP showed a significantly enhanced apoptosis. Since the treatment with the EGF alone did not induce similar degree of apoptosis, we suppose that the conjugated GNP was retained more in the treated cell than unmodified GNP control. However, quantitative determination of internalized GNP and the response to NTP still await further studies.

Mechanism of GNP endocytosis includes clathrin/caveloar-mediated endocytosis, phagocytosis, macropinocytosis, and pinocytosis^45^. It is speculated in this report that the internalization of the GNP was facilitated by EGF conjugation, implying that receptor-mediated endocytosis played an important role. It is already reported that the internalized GNP confers radio-sensitivity on treated cells to increase effect of the ionizing radiation^46^. Thus we also think that the endocytosed GNP in cancer cells could elicit a massive apoptotic response by the NTP irradiation and finally induce cancer regression. We also suggest that this target-specificity of the EGF-GNP conjugates could aim cancer cells expressing high level of the EGFR such as breast cancer^47^.

In this report, we detected a significant elevation of NF-κB expression through the treatment with the EGF-GNP complexes without the NTP irradiation. Since it is previously reported that nanoparticles elicit various cellular responses including inflammation^33^, it can be expected that the EGF-GNP conjugate made the treated cells permissive to apoptosis by expressing the NF-κB. Indeed, there was no apoptosis with the treatment with the EGF-GNP conjugates without NTP irradiation at Fig. 3D. However, 50 seconds of NTP irradiation at 2.5 kV showed marked decrease in cell viability after the treatment with the EGF-GNP as shown in Fig. 4A. Thus, it seems like that the treatment with the EGF-GNP conjugate alone is not appropriate to elicit apoptosis of treated cells.

The present study shows that the EGF-conjugated GNP was internalized into a cell through EGFR-mediated endocytosis. Subsequent irradiation of NTP could effectively elicit a specific apoptotic response in GNP-treated cells. Taken together we suggest that this novel strategy we introduced here may overcome current limitations of plasma medicine in cancer treatment.

## Methods

### Cell culture

A549, DU145, HT29, and SK-OV(3) cell lines were grown in RPMI-1640 medium (Hyclone, Pittsburgh, PA). SH-SY5Y and HeLa cell lines were grown in Dulbecco’s Modified Eagle Medium (DMEM) (Hyclone, Pittsburgh, PA). Both media contained 10% fetal bovine serum (FBS) (Hyclone, Pittsburgh, PA) and 1% penicillin/streptomycin (Sigma-Aldrich, ST. Louis, MO). Cells were incubated under humidified atmosphere with 5% CO_2_ at 37 °C. For serum starvation, A549 cells were cultured in RPMI-1640 medium without serum, complemented with 1% penicillin/streptomycin for 24 hours at 37 °C. Cells were counted after stained with 0.4% trypan blue solution (Sigma-Aldrich, St. Louis, MO).

### EGF conjugation with 5 nm-gold nanoparticle (GNP)

The EGF-conjugated GNP was prepared as we previously described^21^. Briefly, 0.1 mg/ml aqueous solution of 3-mercaptopropionic acid (MPA), 11-mercaptoundecanoic acid (MUA), and 16-mercaptohexadecanoic acid (MHDA) were prepared and incubated with 5 nm colloidal gold (Sigma-Aldrich, St. Louis, MO) suspension. After overnight incubation at room temperature, mixtures were reacted with 1 mM N-ethyl-N′-(3-dimethylaminopropyl) carbodiimide (EDC) solution (Sigma-Aldrich, St. Louis, MO) for 30 minutes at room temperature. EDC-terminated gold nanoparticle was incubated with 1 mg/ml of EGF peptide. After centrifugation, 100 μl of 1% BSA (Bovine Serum Albumin) was added and stored at 4 °C.

### Western, dot blotting, and antibodies

Harvested cells were lysed in buffer containing 4% SDS and 2 M urea in phosphate-buffered saline (PBS). Western blotting and dot blotting were performed as we previously described^48^. Antibodies were purchased as indicated: anti-EGFR (Abcam, Cambridge, MA), anti-GAPDH (ICN Biomedicals, Irvine, CA), anti-phospho-ERK (Santa Cruz Biotechnology, Dallas, TX), anti-14-3-3ξ (Santa Cruz Biotechnology, Dallas, TX), anti-EGF (Abcam, Cambridge, MA), anti-BAX (Cell Signaling Technology, Danvers, MA), anti-p53 (Santa Cruz Technology, Dallas, TX), anti-γH2AX (Cell Signaling Technology, Danvers, MA). HRP-conjugated species-specific secondary antibodies (KPL, Gaithersburg, MD) were visualized under LAS-4000 chemiluminescence detection system (FUJIFILM, Tokyo, Japan). Acquired images were analyzed using Image Gauge (FUJIFILM, Tokyo, Japan) according to the manufacturer’s instruction.

### Immunocytochemistry

A549 cells were seeded on cover glasses in RPMI-1640 medium (Hyclone, Pittsburgh, PA) at 37 °C or 29.5 °C in a humidified incubator under 5% CO_2_. Treated cells were washed with PBS, and fixed with 4% paraformaldehyde (PFA) in PBS for 20 min. 2% NP-40 in PBS was used to permeabilize cells for 5 min at room temperature. Cells were next incubated with blocking solution containing 10% FBS in PBS for 30 minutes at room temperature. Anti- EGFR (Abcam, Cambridge, MA) antibody in blocking solution was applied and, the protein was visualized with Alexa 488-conjugated secondary antibody. Nuclear counter staining was performed with 2 μg/ml of Hoechst 33342 (Sigma-Aldrich, St. Louis, MO). Images were captured by Axioplan2 fluorescence microscope (Zeiss, Oberkochen, Germany).

### Assessment of cell viability and apoptosis

Cell viability was assessed by using a chromogenic assay involving biological reduction of the tetrazolium salt 3-(4,5-dimethylthiazol-2-yl)-2,5-diphenyltetrazoliumbromide (MTT), which is converted into blue formazan crystals by living cells. A549 cells were seeded in 96-well plates and cultured as indicated. 12.5 μl of 10 mg/ml MTT solution (Sigma-Aldrich, St. Louis, MO) was added and incubated for 2 hours at 37 °C. 100 μl of solubilizing solution (25% of SDS, 62.5% of dimethyl formamide) was added and incubated for 3 hours at 37 °C. Optical density was measured at 570 nm.

### TUNEL (Terminal deoxynucleotidyl transferase dUTP nick end labeling) assay

A549 cells were seeded on cover glasses in RPMI-1640 medium. Cells were washed with PBS and fixed with 4% paraformaldehyde for 20 minutes at room temperature. 0.5% NP-40 in PBS was used to permeabilize the cells. To detect damaged DNA, DeadEND^TM^ Fluorometric TUNEL System (Promega, Madison, WI) was used according to the manufacturer’s instruction. Briefly, equilibration buffer was applied to cover glass, followed by addition of nucleotide mix and rTdT enzyme. After 1 hour of incubation at 37 °C, cells were washed and counterstained.

### Data availability

The datasets generated during and/or analyzed during the current study are available from the corresponding author on reasonable request.
